# Genetic Screening for Potential New Targets in Chronic Myeloid Leukemia Based on Drosophila Transgenic for Human BCR-ABL1

**DOI:** 10.3390/cancers13020293

**Published:** 2021-01-14

**Authors:** Marco Lo Iacono, Elisabetta Signorino, Jessica Petiti, Monica Pradotto, Chiara Calabrese, Cristina Panuzzo, Francesca Caciolli, Barbara Pergolizzi, Marco De Gobbi, Giovanna Rege-Cambrin, Carmen Fava, Claudia Giachino, Enrico Bracco, Giuseppe Saglio, Francesco Frassoni, Daniela Cilloni

**Affiliations:** 1 Department of Clinical and Biological Sciences, University of Turin, 10043 Turin, Italy; elisabetta.signorino@unito.it (E.S.); jessica.petiti@unito.it (J.P.); monica.pradotto@gmail.com (M.P.); chiara.calabrese@unito.it (C.C.); cristina.panuzzo@unito.it (C.P.); francesca.caciolli@unito.it (F.C.); barbara.pergolizzi@unito.it (B.P.); marco.degobbi@unito.it (M.D.G.); giovanna.rege@libero.it (G.R.-C.); carmen.fava@unito.it (C.F.); claudia.giachino@unito.it (C.G.); giuseppe.saglio@unito.it (G.S.); francesco.frassoni19@gmail.com (F.F.); daniela.cilloni@unito.it (D.C.); 2 Department of Oncology, University of Turin, 10043 Turin, Italy; enrico.bracco@unito.it

**Keywords:** chronic myeloid leukemia, BCR-ABL1, Rab family, *Drosophila melanogaster*, genetic screening

## Abstract

**Simple Summary:**

Chronic myeloid leukemia (CML) is a myeloproliferative neoplasm characterized by the presence of the Philadelphia chromosome that encodes for the tyrosine kinase protein BCR-ABL1 from the *Breakpoint Cluster Region* (*BCR*) sequence and the *Abelson* (*ABL1*) gene. Despite BCR-ABL1 being one of the most studied oncogenic proteins, several of its partners, acting either as positive or negative BCR-ABL1 regulators, are still unknown. To identify the new components involved in BCR-ABL1 transforming activity, we conducted an extensive genetic screening using different Drosophila mutant strains. We identified several putative candidate genes that may be involved either in sustaining CML or in its progression. For the first time, we identified in Drosophila and confirmed in CML patients a tight connection between the BCR-ABL1 protein and Rab family members. Our data strongly suggest that Drosophila is a powerful tool to dissect BCR-ABL1 etiology and to identify unknown pathways, thus providing the basis for the development of new potential therapeutic strategies for the treatment of CML patients.

**Abstract:**

Chronic myeloid leukemia is a myeloproliferative neoplasm characterized by the presence of the Philadelphia chromosome that originates from the reciprocal translocation t(9;22)(q34;q11.2) and encodes for the constitutively active tyrosine kinase protein BCR-ABL1 from the *Breakpoint Cluster Region* (*BCR*) sequence and the *Abelson* (*ABL1*) gene. Despite BCR-ABL1 being one of the most studied oncogenic proteins, some molecular mechanisms remain enigmatic, and several of the proteins, acting either as positive or negative BCR-ABL1 regulators, are still unknown. The Drosophila melanogaster represents a powerful tool for genetic investigations and a promising model to study the BCR-ABL1 signaling pathway. To identify new components involved in BCR-ABL1 transforming activity, we conducted an extensive genetic screening using different Drosophila mutant strains carrying specific small deletions within the chromosomes 2 and 3 and the *gmrGal4,UAS-BCR-ABL1 4M/TM3* transgenic *Drosophila* as the background. From the screening, we identified several putative candidate genes that may be involved either in sustaining chronic myeloid leukemia (CML) or in its progression. We also identified, for the first time, a tight connection between the BCR-ABL1 protein and Rab family members, and this correlation was also validated in CML patients. In conclusion, our data identified many genes that, by interacting with BCR-ABL1, regulate several important biological pathways and could promote disease onset and progression.

## 1. Introduction

Chronic myeloid leukemia (CML) is a myeloproliferative neoplasm characterized by the presence of the Philadelphia chromosome (Ph), which originates from the reciprocal translocation t(9;22)(q34;q11.2). This translocation results in a fusion oncogene between the *Breakpoint Cluster Region* (*BCR*) sequence and the *Abelson* (*ABL1*) gene, which encodes for the constitutively active tyrosine kinase protein BCR-ABL1 [1,2]. Among the different isoforms described for the BCR-ABL1 fusion protein, the p210 (210-KDa protein) is the most common in CML patients. ABL1 is a nonreceptor tyrosine kinase ubiquitously expressed and usually located in the nucleus. The fusion with BCR moves ABL1 to the cytoplasm, enabling it to interact with several proteins and exert its leukemogenic effect. The fusion protein BCR-ABL1 becomes constitutively active and, through autophosphorylation, generates several binding sites for proteins that possess an SH2 domain [3]. The leukemogenic pathway activated by BCR-ABL1 has been linked to changes in growth factor dependence, apoptosis, proliferation, and cell adhesion, causing the uncontrolled proliferation of granulocytes in the initial stages of the disease [4,5,6,7,8,9]. Despite BCR-ABL1 being one of the most studied oncogenes, most of the proteins, acting as positive or negative regulators of BCR-ABL1, are still unknown. Indeed, some mechanisms underlying the inexorable progression of the disease remain enigmatic.

The fruit fly, *Drosophila melanogaster*, represents a powerful tool for genetic investigations and a promising model to study the BCR-ABL1 signaling pathway through the identification of genes encoding for proteins that, to a different extent, contribute to the BCR-ABL1 phenotype. The simplicity of genetics and ease of manipulation in *Drosophila* present it as an attractive model. In addition, Drosophila hematopoiesis is comparable to that of mammals, and the high homology levels between human and *Drosophila* genes are important advantages of using *Drosophila* to understand human diseases [10]. This allowed conducting an extensive genetic screening to identify genes that eventually may modulate the phenotype that characterizes the disease under investigation [11,12]. Indeed, a *Drosophila* model for t(8;22) was used to successfully identify new modulators of the AML1-ETO fusion protein [13,14]. In order to identify new BCR-ABL1 partners, we previously generated and validated a *Drosophila* model of CML suitable to perform genetic screening to fish out genes potentially involved in CML onset and/or progression [15]. In our model, the expression of the BCR-ABL1 oncoprotein in eye cells induces a strong phenotype characterized by altered differentiation of the ommatidia cells. In this work, we conducted an extensive genetic screening using different available strains of *Drosophila* carrying specific small deletions for chromosomes 2 and 3 and our *gmrGal4,UAS-BCR-ABL1 4M/TM3* transgenic *Drosophila* as “bait”. By observing the changes in the phenotype, it was possible to identify several candidate genes in different pathways that may be involved in CML sustaining and progression.

## 2. Results

### 2.1. Drosophila BCR-ABL1 Phenotype Was Altered Upon Deletion Stocks Crossing

**Figure 1:**
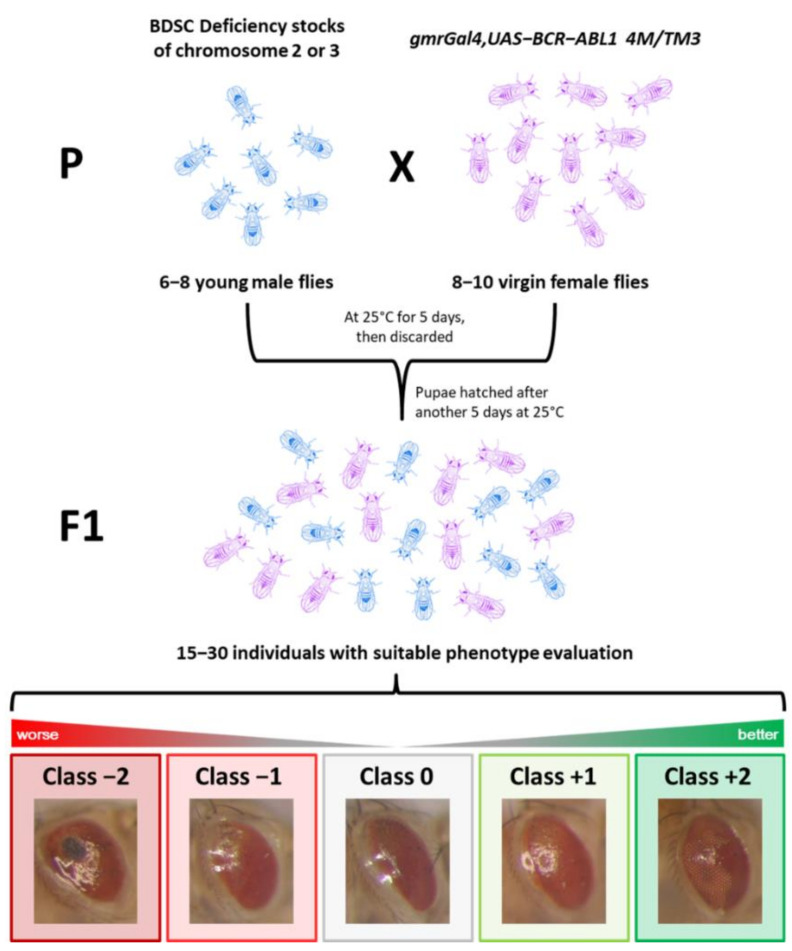
Typical crossing set up for BCR-ABL1 (from the *Breakpoint Cluster Region* (*BCR*) sequence and the *Abelson* (*ABL1*) gene) screening and phenotypical classes. Schematic view of crossing between male flies harboring deletions on chromosomes 2 and 3 and virgin female flies carrying BCR-ABL1 transgene expressed in the eyes of the animals (genotype *gmrGal4,UAS-BCR-ABL1 4M/TM3*). Typically, 6–8 young males are chosen from the deficiency stock and put together with 10 virgin females *gmrGal4,UAS-BCR-ABL1 4M/TM3* in a new tube (P generation), left at 25 °C for 5 days, and then discarded. After another 5 days at 25 °C, pupae start hatching, and 15–30 F1 generation individuals with the suitable phenotype are photographed and classified. F1 flies (15–30) from three independent crosses with the suitable genotype were classified into five arbitrary phenotypic classes. Class 0 (gray) represents the average phenotype of *gmrGal4,UAS-BCR-ABL1 4M* flies, class −1 (light red) represents a stronger eye phenotype, class −2 (dark red) the strongest eye phenotype, class +1 (light green) represents a weaker phenotype, and class +2 (dark green) represents the weakest phenotype. BDSC: Bloomington Drosophila Stock Center.

This work aimed to screen the genome of *Drosophila* looking for positive and negative regulators of the BCR-ABL1 oncogenic protein that may be involved in the sustaining and progression of CML. Using a recombinant fly carrying both the *gmrGal4* and the UAS-BCR-ABL1 transgenes in *cis* on the third chromosome (*gmrGal4,UAS-BCR-ABL1 4M/TM3*), we were able to perform a genetic screening of most of the *Drosophila* genome (autosomes 2 and 3). This was performed by crossing the BCR-ABL1 recombinant fly with more than 200 stocks of deletions (Figure 1 and Table S1) covering approximately 10,000 *Drosophila* genes and by classifying the changes of the resulting phenotypes in five arbitrary classes: class 0 represents the average BCR-ABL1 phenotype; class +1 represents a weaker phenotype, characterized by an improvement of either dimension or differentiation; class +2 represents the weakest phenotype, with an improvement in both dimension and differentiation with at least a partial rescue of phenotype; class −1 represents a stronger phenotype, characterized by a worsening of either dimension or differentiation; and class −2 represents the strongest phenotype, characterized by a worsening of both dimension and differentiation, the presence of necrosis areas, and more misplaced extrasensory bristles (Figure 1, Figure S1 and Table S1). The eye phenotype is not always completely penetrant, but here, we chose to report only the most represented class for each cross.

**Figure 2:**
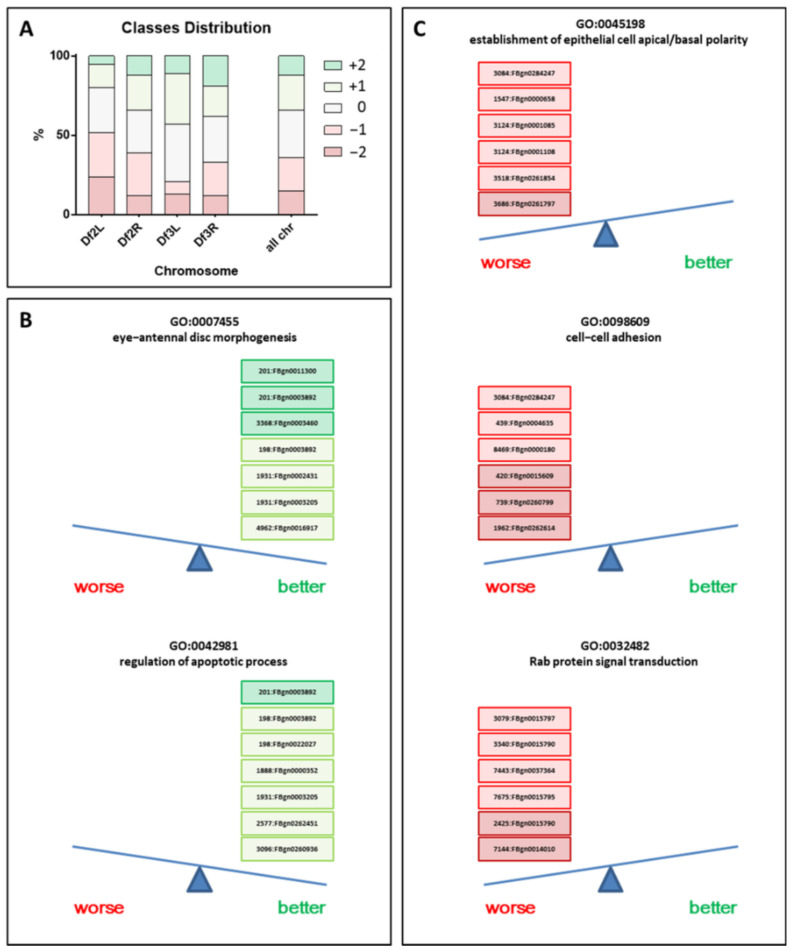
Classification of BCR-ABL1 phenotype modifications and top gene ontology (GO) classes. (**A**) Schematic representation of the distribution of phenotype modifications among the chromosome arm deficiency stocks analyzed (Df2L, Df2R, Df3L, and Df3R). (**B**) The GO classes with better stock/gene frequencies that partially rescued the eye phenotype: 7 stocks with +1 or +2 vs. 0 with −1 or −2 classes. (**C**) The GO classes with better stock/gene frequencies that worsened the eye phenotype: 0 stocks with +1 or +2 vs. 6 with −1 or −2 classes.

Out of 217 stocks of deletions, we found a total of 58 major modifiers, 33 (16%) belonging to class +2 and 25 (12%) belonging to class −2. Furthermore, we found 90 minor modifiers, 43 (20%) classified as +1 and 47 (22%) as −1. A total of 63 stocks were classified as nonmodifiers (class 0, 30%) (Figure 2A and Table S1). In particular, the third chromosome accounted for the major presence of pejorative phenotypes (−2 and −1 class): 43% in left arm (L) and 38% in right arm (R) compared to chromosome 2, which accounted for 20% and 34% in L and R, respectively. In addition, the chromosome 2L showed the maximal presence of an ameliorative phenotype (+2 and +1 class): 52% with respect to 39% of 2R and 33% of 3L and 3R, respectively (Figure 2A and Table S1). After the removal of those deletions that did not affect the original phenotype, all results were clustered according to the “biological process” gene ontology (GO) term, and the results are summarized in Table S2. Stocks and genes with the highest frequency in the “biological process” GO classes for the ameliorative (seven stocks with +1 or +2 vs. zero with −1 or −2 classes) and pejorative phenotypes (zero stocks with +1 or +2 vs. six with −1 or −2 classes) are shown in Figure 2B,C, respectively. Interestingly, in some of these GO families, genes identified in more than one stock were present, such as FBgn0003892, included in all ameliorative top classes and identified in two different stocks #201 and #198, or FBgn0015790, included in two pejorative stocks (#2425 and #3340) for the “Rab protein signal transduction” GO class.

### 2.2. Rab Genes as a New Potential Family Involved in Pathological BCR-ABL1 Mechanism

**Figure 3:**
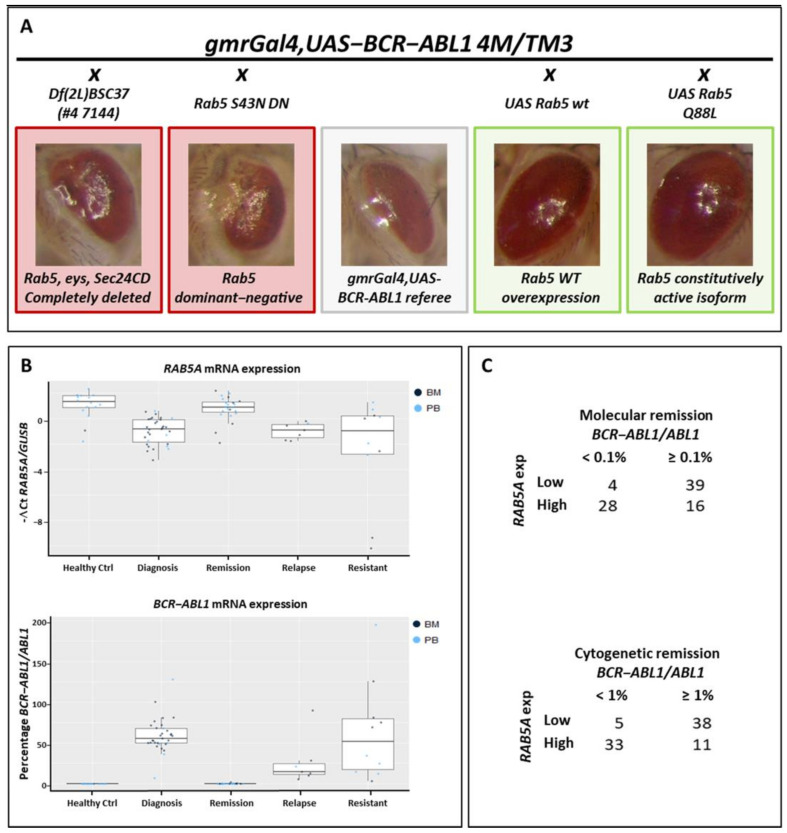
Rab genes as a new potential family involved in the pathological BCR-ABL1 mechanism. (**A**) BCR-ABL1 transgenic flies crossed with 3 different stocks of *Drosophila* bearing different mutated *Rab5*. *Rab5.S43N DN* expressing a dominant-negative isoform of the protein, *UAS Rab5 wt* overexpressing the wild-type isoform, and *UAS Rab5.Q88L* encoding a constitutively active isoform. Class 0 (gray) represents the average phenotype of *gmrGal4,UAS-BCR-ABL1 4M* flies, class −2 (dark red) represents the strongest eye phenotype, and class +1 (light green) represents a phenotype. (**B**) Modulations of *RAB5A and BCR-ABL1* were analyzed with a *t*-test in chronic myeloid leukemia (CML) patients at different stages of the disease and in healthy controls. BM, bone marrow and PB, peripheral blood. (**C**) Relationship between the *RAB5A* expression levels and *BCR-ABL1/ABL1* percentage. *RAB5A* was dichotomized based on the median value, while *BCR-ABL1/ABL1* was stratified by molecular (upper panel) and cytogenetic (bottom panel) remission.

Concerning all GO observed in Figure 2 that involved large families (“regulation of apoptotic process” and “cell–cell adhesion”), the GO:0032482 class stands out for a gene pathways specificity: “Rab protein signal transduction”. The genes identified in this family include: FBgn0015797 (Rab6), FBgn0014010 (Rab5), FBgn0015795 (Rab7), FBgn0037364 (Rab23), and FBgn0015790 (Rab11), found in two independent stocks. Intriguingly, compared to all the other stocks in this family that affect several genes, only three genes were completely destroyed in stock #7144: Rab5, eys, and Sec24CD. Furthermore, F1 generation obtained by crossing the #7144 stock and *gmrGal4,UAS-BCR-ABL1 4M/TM3* showed a particularly severe worsening of the BCR-ABL1 phenotype (Figure 3A, left red-framed, and Figure S2). Since the stock with the deletion of *Rab5* carried the deletion of only two other genes, we decided to investigate the weight of *Rab5* in the observed phenotype. Therefore, we decided to study the impact of *Rab5* modulation on the BCR-ABL1 phenotype. We crossed the BCR-ABL1 transgenic flies with three different stocks of *Drosophila* harboring different *Rab5* forms. In detail, we used one stock of *Rab5* expressing a dominant-negative isoform of the protein (*Rab5.S43N DN*), one overexpressing the wild-type isoform (*UAS Rab5 wt*), and one encoding a constitutively active form (*UAS Rab5.Q88L*). The eyes of the flies co-expressing BCR-ABL1 and the dominant-negative (DN) of *Rab5* showed a marked worsening of the BCR-ABL1 phenotype, affecting the normal round morphology and pigmentation and showing areas of necrosis (Figure 3A, right red-framed). Furthermore, the co-expression of BCR-ABL1 and the overexpression of either the wild-type allele or the constitutively active isoform of *Rab5* partially rescued the BCR-ABL1 phenotype (Figure 3A, green-framed). Supported by the promising data obtained by our screening, we decided to evaluate the mRNA expression levels of the *RAB5A* gene, the principal human ortholog of *Drosophila Rab5*, in healthy controls and CML patients. To this aim, we collected several peripheral blood (PB) and bone marrow (BM) samples from patients at different stages of the disease: at diagnosis, during the cytogenetic or molecular response, and during the secondary resistance to tyrosine kinase inhibitors (TKI). In addition, another 10 specimens were collected from patients with a primary resistance to TKI. The results were presented in Figure 3B. Interestingly, the qRT-PCR analysis revealed a significant downregulation of *RAB5A* in CML patients at diagnosis (Diagnosis, mean −0.98; CI 1.34–2.69; *t*-test *p* << 0.01), during secondary resistance (Relapse, mean −0.97; CI 1.25–2.77; *t*-test *p* << 0.01), and in primary resistant subjects (Resistant, mean −2.46; CI 0.53–6.46; *t*-test *p* = 0.02) compared to healthy controls (mean +1.04) (Figure 3B, upper panel). In contrast, when we observed cytogenetic or molecular remission, the expression of *RAB5A* increased, achieving the same levels observed in healthy donors (Remission, mean +0.78; CI −0.43–0.95; *t*-test *p* = 0.44). This pattern was also observed when we stratified specimens by sample types (BM or PB). Our data suggested an inverse correlation between *RAB5A* expression and the presence of a *BCR-ABL1* transcript, highlighted by their opposite behavior (Figure 3B). To investigate this association, we dichotomized the *RAB5A* expression and evaluated the correlation with the *BCR-ABL1* transcript, subdivided using two clinical diagnostic *BCR-ABL1* levels thresholds: cytogenetic (<1%) and molecular remission (<0.1%). As indicated by Figure 3C, we observed significant opposite regulations of these two transcripts, suggesting a direct downmodulation of *RAB5A* in the presence of the Philadelphia chromosome (cytogenetic: *p* << 0.01; odds ratio 0.05; CI 0.01–0.15 and molecular: *p* << 0.01; odds ratio 0.06; CI 0.01–0.21). This data was consistent with what we observed in *Drosophila*, meaning *RAB5A* and/or its family could potentially be new targets involved in BCR-ABL1 pathological signaling.

## 3. Discussion

Chronic myeloid leukemia is a myeloproliferative neoplasm characterized by the presence of the Philadelphia chromosome. Despite BCR-ABL1 representing one of the most studied oncogenic proteins, some molecular mechanisms leading to cellular transformation are still partially unknown, and its positive or negative regulators are not completely identified. The fruit fly, *Drosophila melanogaster*, represents a powerful tool for a genome-wide genetic analysis and screens, given the functional conservation and sequence homology between human and *Drosophila* genes. In this work, we conducted a genome-wide genetic screening using commercial gene deletion stocks for autosomal chromosomes 2 and 3 and our previously generated and validated CML *Drosophila* model, *gmrGal4,UAS-BCR-ABL1 4M/TM3*, as “bait” [15]. Although deletion stocks include several genes often partially or completely destroyed generating complex phenotypes, we were able to filter out some important information that could enrich the knowledge of the BCR-ABL1 pathological pathway. In particular, we identified two GO families concerning ameliorative phenotypes: “regulation of the apoptotic process” and “cell–cell adhesion”. As members of these two classes, we identified the *patched* gene (FBgn0003892), an ortholog to human *PTCH1* (*patched 1*) and *PTCH2* (*patched 2*). Protein patched homolog 1 (PTCH1) encodes for a cell-surface receptor and is a member of the Hedgehog (Hh) signaling pathway. The PTCH1 protein exerts its function by negatively regulating the activity of the frizzled family receptor smoothened (SMO) [16]. There is evidence that this pathway plays a critical role in the etiology of various hemopoietic malignancies—in particular, in CML [17]. It has been reported in the literature that Hh signaling is increased in BCR-ABL1-positive progenitor cells, and it is further upregulated during the disease progression [18]. Furthermore, *PTCH1* overexpression and mutations in CML patients are associated with poor prognosis and primary or secondary resistance to tyrosine kinase inhibitors [19,20,21,22]. All this data supports our results, showing that the suppression of the *patched* gene generates a better phenotype, suggesting that BCR-ABL1 and patched, when present together, cooperate for the disease etiology. In addition, among genes of “eye-antennal disc morphogenesis”, we found *babo* (FBgn0011300), a gene activated by patched and SMO signaling in *Drosophila* [23]. Intriguingly, *babo* is the ortholog of *TGFBR1* (best score), a representative of the Transforming Growth Factor-β Receptor family (TGF-βR). It is known that the TGF-βR signaling pathway plays an important role in CML, leading to cell growth inhibition, differentiation, and apoptosis [24]. Interestingly, when *patched* and *babo* were simultaneously deleted, as in stock #201, we observed a better phenotype compared to #198, where only *patched* was suppressed. This result suggests the powerful cooperation of these genes with BCR-ABL1, further highlighting that *Drosophila* is a powerful model to identify positive and negative BCR-ABL1 partners. Additionally, in the worst phenotype classes, we identified some genes that could cooperate with BCR-ABL1 in CML. One of these is *frizzled* (FBgn0001085), a seven-pass transmembrane domain receptor, ortholog of human *FZD7*, involved in the Wnt signaling pathway, a gene probably involved in CML cell protection mediated by bone marrow-derived mesenchymal stem cells [25]. Recently published evidence suggests that the atypical G protein-coupled receptors (GPCRs) Frizzled and SMO regulate the Hippo pathway in a G protein-dependent manner and contribute to the crosstalk between Hippo and other important pathways (such as Wnt and Hh) relevant for the development process and carcinogenesis [26]. BCR-ABL1 involvement in these fundamental biological processes was also supported by our data, which identified several additional genes participating in these pathways, such as *aPKC* (FBgn0261854) and *four-jointed* (FBgn0000658). Furthermore, interactions with the cadherins pathway (dachsous (FBgn0284247), Cadherin-N (FBgn0015609), and adherens junction protein p120 (FBgn0260799)) are also supported by data of the literature. Indeed, Zang et al. reported that N-cadherin expression contributes to increased resistance to farnesyltransferase inhibitor SCH66336 in BCR-ABL1^p190^-positive acute lymphoblastic leukemia [27]. Furthermore, Mu et al. identified that the expression of *CDH13*mRNA in CML patients is lower than in healthy subjects, showing a negative correlation with the *BCR-ABL1* fusion gene that may contribute to CML development [28]. 

Taken together, these results suggest the usefulness of our screening system and, therefore, give us confidence in the goodness of the data we have obtained for new possible modulators that have never been described before. In particular, in our study, we identified for the first time a tight connection between the BCR-ABL1 protein and Rab family members (Rab5 (FBgn0014010), Rab6 (FBgn0015797), Rab7 (FBgn0015795), Rab11 (FBgn0015790), and Rab23 (FBgn0037364)) and between BCR-ABL1 and the class of microtubule-based kinesin and cytoplasmic dynein motor complexes that are the main Rab effectors (Dhc64C (FBgn0261797) and DCTN1-p150 (FBgn0001108)). The Rab GTPase proteins were first studied in yeast *Saccharomyces cerevisiae* and are the master regulators of all stages of the intracellular trafficking processes in eukaryotic cells evolutionarily conserved from fly to vertebrates. The *RAB5* gene, which encodes for a monomeric small GTPase, is a key member of the Rab family, and *RAB5A* is its most important subtype, with well-identified functions and mechanisms. RAB5A affects the internalization and intracellular transport of receptors, such as receptor tyrosine kinases, GPCRs, and antigen-recognition receptors, by recruiting Rab5 effectors. The signals mediated by RAB5A range between gene transcription, cell morphology, growth, differentiation, apoptosis, and disease development [29]. In our analysis, we observed a worsening of the BCR-ABL1-induced phenotype in the absence of Rab5 expression, and these data were consistent with the results obtained with other members of the Rab family identified by our screening. This scenario was also confirmed in primary cells from CML patients, where the expression of the *RAB5A* transcript was significantly reduced in leukemic cells while returned to normal levels during the remission phase. In addition, we identified a significant opposite regulation of *BCR-ABL1* and *RAB5A* mRNA. The *BCR-ABL1* transcript, when present, seems to downmodulate *RAB5A* expression, probably in order to acquire some molecular advantages, such as the reduction of receptor turnover and the increase of the proliferative stimuli. In support of this hypothesis, we previously identified that the downregulation of Rab interactor 1 (RIN1) and Bridging integrator 1 (BIN1), two proteins directly involved with Rab-mediated receptor tyrosine kinase intracellular trafficking, caused aberrant and constitutive receptor signaling and are often observed deregulated in CML-resistant patients [30]. A plausible hypothesis regarding this result could be that the decrease of RAB5A gives an advantage in terms of a longer permanence in the membrane of BCR-ABL1. Although further studies are needed to verify its validity, this hypothesis is supported by previous studies that demonstrated how the Rab family can regulate the signals mediated by different membrane proteins through internalization and recycling. Indeed, it has been shown that the Rab family can regulate the EGFR signal through degradation and recycling mediated by the endocytic pathway [31]. Successively, Bastin and Heximer showed that both RAB5 activation and RAB11 inhibition decreased RGS4 function, describing, for the first time, Rab GTPase’s involvement in the intracellular trafficking of an RGS protein [32]. In a complex scenario such as that of CML, it is plausible to think that decreased vesicular traffic can increase the activity of different membrane receptors, thus supporting the proliferative stimuli of myeloid cells.

We are aware of the limitations of our artificial system that could include different “non-gene-specific” deletions on chromosomes 2 and 3, the interactions between proteins of different species, and eye-limited protein expression. Some of these were anyway shared with other drosophila models, such as AML-ETO1, for which different modulators were successfully identified [13,14]. In addition, the encouraging results, obtained by the categories already associated with BCR-ABL1 and the newly described Rab family, could represent a starting point to dissect the contribution of other BCR-ABL1 putative modulators here identified in the etiology of CML (Table S2). We know that further specific studies will be necessary to validate our findings molecularly and clinically in CML patients.

## 4. Materials and Methods

### 4.1. Drosophila Stocks and Screening

Fly stocks of drivers and deletions were obtained from the Bloomington Drosophila Stock Center (BDSC) (Department of Biology, Indiana University, Bloomington, IN, USA), and they are described at FlyBase (https://flybase.org/). *BCR-ABL1* transgenic flies were generated as previously described [15]. To perform the genetic screening, flies from a recombinant line carrying both the *gmrGal4* and the *UAS-BCR-ABL1* transgenes in *cis* on the 3rd chromosome (*gmrGal4,UAS-BCR-ABL1 4M/TM3*) were crossed with lines carrying different deletions on the second (DK2L and DK2R) and third chromosomes (DK3L and DK3R). This resulted in an eye phenotype that can be easily and quickly scrutinized. To evaluate the phenotype, 15–30 first-generation (F1) flies, from three independent crosses, were classified into five arbitrary phenotypic classes. Class 0 represents the average BCR-ABL1 phenotype: the eye was smaller, bar-shaped, and misplaced extra sensory bristles appeared in the dorsal region. In addition, ommatidia were no longer distinguishable. Class +1 represents a weaker phenotype, characterized by an improvement of either dimension or differentiation. Some disorganized ommatidia could be present. Class +2 represents the weakest phenotype, with an improvement in both dimension and differentiation, with at least a partial rescue of the phenotype. Indeed, large regions of organized ommatidia were observed, although the eye was not completely developed. Class −1 represents a stronger phenotype, characterized by a worsening of either dimension or differentiation. Some lack of pigmentation and sporadic very small necrosis areas were observed in the eye. Class −2 represents the strongest phenotype, characterized by a worsening of pigmentation, dimension, and differentiation. This phenotype was characterized by the presence of large necrosis areas and more misplaced extrasensory bristles. In addition, we analyzed also the following BDSC Rab5 stocks: Rab5.S43N (#9771), UAS Rab5 wild type (#24616), and UAS Rab5.Q88L (#9773). All flies were cultured in standard medium and grown at 25 °C, if not otherwise specified. To examine the eye phenotypes, images of adult eyes were captured using a Leica stereomicroscope 5.7× equipped with a photo-camera (Olympus, Olympus Italia Srl, Segrate (MI), Italy).

### 4.2. Samples from CML Patients

Eighty-seven bone marrow aspirate (BM) and peripheral blood (PB) samples from 56 CML patients and 16 healthy subjects were collected. The study was approved by the San Luigi Hospital Institutional Review Board (approval number 212/2015). All the patients included in the study signed written informed consent. Thirty-one samples were collected at diagnosis, 23 during cytogenetic or molecular remission, and 7 during secondary resistance to tyrosine kinase inhibitors (TKI). An additional 10 specimens were collected from patients with a primary resistance to TKI. White blood cells were separated by buffy coat, and total RNA was extracted using standard procedures. Expression levels of *RAB5A* and the housekeeping gene *GUSB* were evaluated by Real-Time PCR (qRT-PCR) using specific primers and probes assays (Hs00939627_m1 for *GUSB* and Hs00991290_m1 for *RAB5A,* Applied Biosystems and Thermo Fisher Scientific, Waltham, MA, USA) according to the published methods [33].

### 4.3. Bioinformatics and Statistical Analysis

All genes completely or partially disrupted/deleted included in each *Drosophila* stock were reannotated by a homemade Perl script using updated information from the Bloomington *Drosophila* Stock Center (https://bdsc.indiana.edu/) and FlyBase (https://flybase.org/). Each stock was annotated in the appropriate phenotypic class (−2, −1, 0, +1, or +2). To highlight the genes implied in the phenotype changes, we filtered with the following criteria: For each stock, we removed from the list of deleted genes those that (a) were concomitantly present in class 0, because they were plausibly not altering the phenotype and (b) those that were found concurrently in opposed phenotypic classes (e.g., −2 and +1). Subsequently, the remaining genes were annotated and sorted based on their frequency of the gene ontology “biological process” in ameliorative (+1 and +2 class) or pejorative phenotypes (−1 and −2 class) (Table S2).

The *RAB5A*mRNA cycle threshold values (CtT) were calculated by Bio-Rad CFX manager 3.1 software and normalized against the internal control *GUSB* (CtR) by subtraction (CtT-CtR). ΔCt values generated were then standardized, with ΔCt obtained by universal reference RNA (Stratagene), evaluated in the same run. Differential *RAB5A* transcript expression between −ΔCt values of healthy controls and samples derived from each different clinical subgroup or between groups were evaluated using the *t*-test. *RAB5A*mRNA expression levels were dichotomized into two groups of “high” and “low” expression using the median value as the threshold cut-off. The *BCR-ABL1* transcript presence, evaluated as the percentage of *BCR-ABL1/ABL1*, was dichotomized into groups using two diagnostic criteria: molecular remission (<0.1%) and cytogenetic remission (<1%). The association between the *RAB5A* and *BCR-ABL1* transcripts was evaluated using the Fisher exact test. Statistical analysis was performed using R statistical software (version 3.5.3, Vienna, Austria. URL http://www.R-project.org/).

## 5. Conclusions

The power and the novelty of our model was the possibility to highlight modulators that do not physically interact with BCR-ABL1, and we identified several new putative pathways affecting the BCR-ABL1 phenotype. These molecules regulate several important biological pathways and could promote CML onset and/or progression. Among these, we showed, for the first time, that several members of the RAB family were associated with BCR-ABL1 signaling. Our model also identified several pathways already known for their role in TKI resistance or in CML blast crisis, and this reinforces the strength of our findings. In conclusion, the *Drosophila* model identified still unknown pathways, thus providing the basis for the development of new potential therapeutic strategies for the treatment of CML patients in line with the findings recently published by Outa et al. [34]. Although additional studies are required to individually validate the role of the molecules identified in this work, the *Drosophila* appeared to be a powerful tool to dissect the BCR-ABL1 etiology.

## Data Availability

Data is contained within the article or supplementary material.

## Supplementary Materials

The following are available online at https://www.mdpi.com/2072-6694/13/2/293/s1, Figure S1: Phenotypical classes, Figure S2: #7144 phenotype, Table S1: BDSC stocks and relative phenotypes, Table S2: BDSC stocks, GO and classes.
