# Supplementary material for: Genetic Screening for Potential New Targets in Chronic Myeloid Leukemia Based on Drosophila Transgenic for Human BCR-ABL1
Source: Cancers (Basel). 2021 Jan 14;13(2):293. doi: 10.3390/cancers13020293 (PMC7830713; doi:10.3390/cancers13020293)
Supplement: Supplementary file 1 [file cancers-13-00293-s001.zip › cancers-1036325-supply/cancers-1036325-supply fig.docx]

Supplementary Materials

Genetic Screening for Potential New Targets in Chronic Myeloid Leukemia Based on Drosophila Transgenic for Human BCR-ABL1

Marco Lo Iacono, Elisabetta Signorino, Jessica Petiti, Monica Pradotto,
Chiara Calabrese, Cristina Panuzzo, Francesca Caciolli, Barbara Pergolizzi,
Marco De Gobbi, Giovanna Rege-Cambrin, Carmen Fava, Claudia Giachino,
Enrico Bracco, Giuseppe Saglio, Francesco Frassoni and Daniela Cilloni


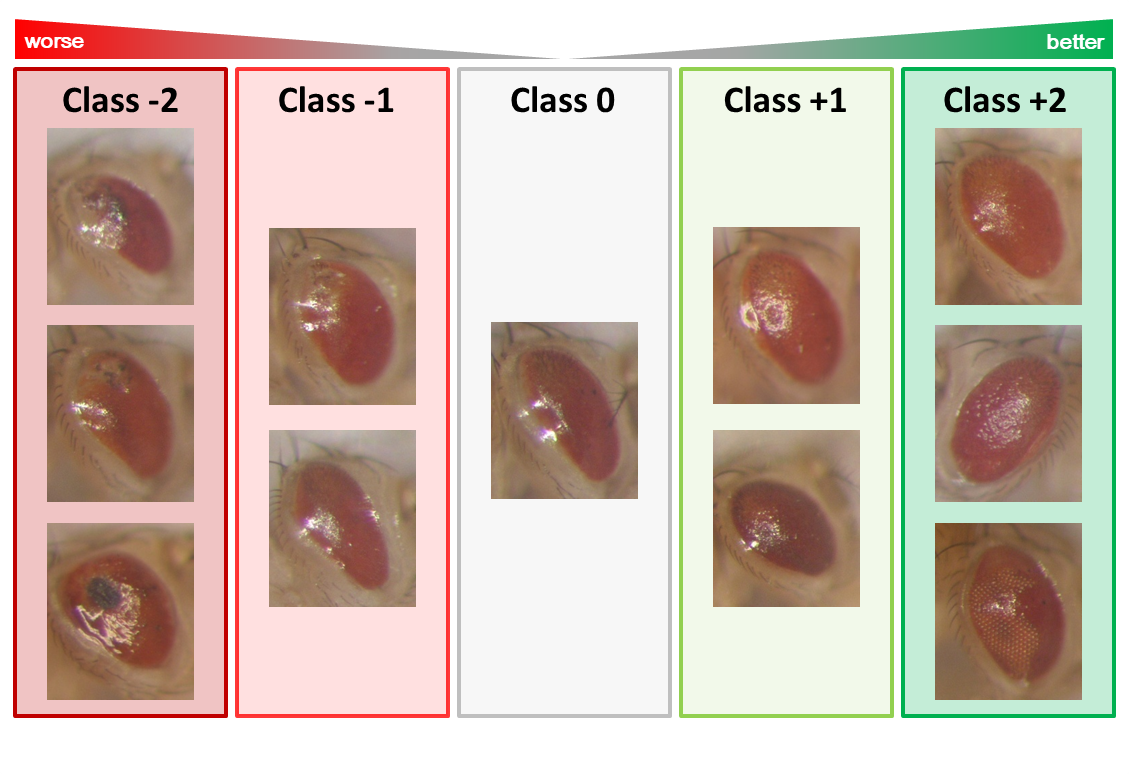


**Figure S1.** Phenotypical classes. Class 0 (gray) represents the average phenotype of *gmrGal4,UAS-BCR-ABL1 4M*  flies; class −1 (light red) represents a more severe eye phenotype and class −2 (dark red) the most severe eye phenotype; class +1 (light green) represents a weaker phenotype and class +2 (dark green) represents the weakest phenotype.


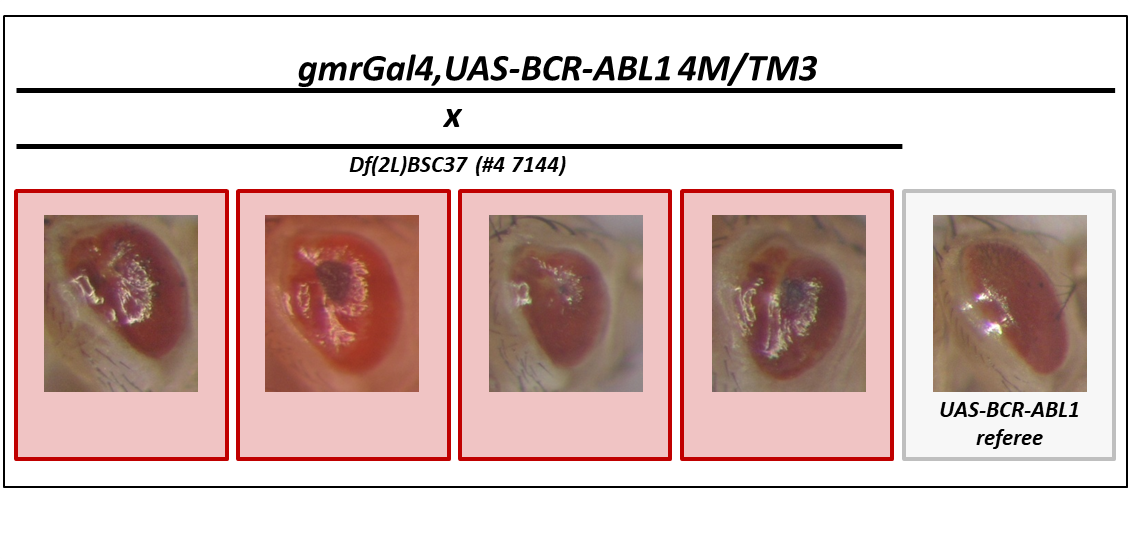


**Figure S2.** #7144 phenotype. Four F1 flies derived by independent crosses between Df(2L)BSC37 #7144 stock and *gmrGal4,UAS-BCR-ABL1 4M/TM3.*

| 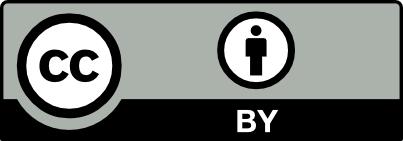 | © 2021 by the authors. Submitted for possible open access publication under the terms and conditions of the Creative Commons Attribution (CC BY) license (http://creativecommons.org/licenses/by/4.0/). |
| --- | --- |
